# Is There Any Role for Super-Extended Limphadenectomy in Advanced Gastric Cancer? Results of an Observational Study from a Western High Volume Center

**DOI:** 10.3390/jcm8111799

**Published:** 2019-10-27

**Authors:** Maria Bencivenga, Giuseppe Verlato, Valentina Mengardo, Lorenzo Scorsone, Michele Sacco, Lorena Torroni, Simone Giacopuzzi, Giovanni de Manzoni

**Affiliations:** 1 General and Upper GI Surgery Division, University of Verona, 3712 Verona, Italy; valentina.mengardo@gmail.com (V.M.); lorenzoscorsone@hotmail.it (L.S.); smichelemed@gmail.com (M.S.); simone.giacopuzzi@univr.it (S.G.); giovanni.demanzoni@univr.it (G.d.M.); 2 Unit of Epidemiology and Medical Statistics, Department of Diagnostics and Public Health, University of Verona, 37134 Verona, Italy; giuseppe.verlato@univr.it (G.V.); lorena.torroni@univr.it (L.T.)

**Keywords:** tailored surgery, gastric cancer, superextended lymphadenectomy

## Abstract

Background: Although the Japan Clinical Oncology Group (JCOG) 9501 trial did not find that prophylactic D3 lymphadenectomy led to any survival advantage over D2 lymphadenectomy, it did find that the prognosis of subserosal and N0 gastric cancer patients improved. The aim of this retrospective observational study was to compare survival after D2 or D3 lymphadenectomy in different patient subgroups. Methods: The study considered all of the patients who underwent D2 or D3 lymphadenectomy at a high-volume center in Verona (Italy) between 1992 and 2011. After excluding patients with Bormann IV or neuroendocrine tumors, early gastric cancers, or non-curative resections, the analysis involved 301 R0 patients: 100 who underwent D2, and 201 who underwent D3 lymphadenectomy. Post-operative deaths and deaths due to recurrences were considered as terminal events in the survival analysis. Results: The D2 patients were significantly older than the D3 patients at baseline (69.8 ± 2.3 vs. 62.2 ± 10.7 years). The median number of retrieved nodes was 29 (interquartile range: 24.5–39) after D2, and 43 (34–52) after D3. The five-year disease-related survival rate was similar after D2 (44%, 95% confidence interval (CI) 34–54%) and D3 (41%, 34–48%) (*p* = 0.766). A Cox model controlling for sex, age, tumor site, Laurén histology, and T and N stages showed that the risk of cancer-related death after D3 was similar to that recorded after D2 (hazard ratio 0.97, 95% CI 0.67–1.42). There was a significant interaction between the T status and the extension of the lymphadenectomy (*p* = 0.012), with the prognosis being better after D2 in T2 and T4b patients, and after D3 in T3 patients. Conclusions: The findings of this study suggest that D3 lymphadenectomy is not routinely indicated for patients with advanced gastric cancer, although differences in survival after D3 across T tiers deserve further consideration.

## 1. Introduction

Gastric cancer is still one of the leading causes of cancer-related deaths worldwide [1]. The only curative treatment is radical resection of the primary tumor and lymph node metastases and, although the optimal extent of lymphadenectomy in gastric cancer has long been debated by Eastern and Western surgeons, D2 lymphadenectomy is currently considered to be the standard of care in almost all of the treatment guidelines [2].

Super-extended lymphadenectomy lost its status as a first-choice treatment for patients with curable gastric cancer after the publication of the Japan Clinical Oncology Group (JCOG) trial, which found no additional survival benefit after D2 plus para-aortic nodes (PAN) dissection in comparison with D2 lymphadenectomy alone [3] in patients with advanced gastric cancer not involving PANs. However, some authors have suggested that PAN dissection may benefit selected groups of advanced gastric cancer patients, as the 5-year survival rate was quite high (18.2%) in the JCOG patients with positive PANs, and similar long-term survival rates have been observed in other Eastern and Western series of patients with positive nodes in nodal station n.16 at pathologic examination (pN16+), including those clinically suspected of being affected by PAN involvement [4,5].

Furthermore, given the satisfactory results of a recent study carried out by the Stomach Cancer Study Group of the Japan Clinical Oncology Group [6], in which patients with locally advanced gastric cancer and extensive regional and/or PAN metastases were treated with neo-adjuvant chemotherapy (S-1 plus cisplatin) followed by extended surgery and PAN dissection (3- and 5-year overall survival rates of 59% and 53%, respectively), Japanese surgeons are now changing their minds about such patients. 

In addition, it needs to be remembered that preoperative N staging in the para-aortic area is not 100% accurate [7], and so some patients may have unrecognized para-aortic metastases.

We believe that identifying subgroups of patients who may benefit from super-extended lymphadenectomy could help surgeons to tailor the extent of nodal dissection on the basis of the tumor’s characteristics because this would not only improve patient survival, but also avoid unnecessary surgical trauma and post-operative complications.

The aim of this observational study was therefore to evaluate whether D3 may be more useful than D2 lymphadenectomy in specific subgroups of patients with advanced gastric cancer by retrospectively reviewing the Upper Gastrointestinal Surgery database of Verona University Hospital.

## 2. Patients and Methods

The study considered all of the 443 patients who underwent D2 or D3 lymphadenectomy for advanced gastric cancer between 1992 and 2011. Twenty-two subjects with Bormann IV tumors or tumors arising from a gastric stump were excluded, as were two subjects with neuroendocrine tumors and 69 with early gastric cancer. Among the remaining patients, 49 had non-curative resection: the proportion of R1 resections was similar in the patients undergoing D2 and D3 lymphadenectomy (6.4% and 8.0%), but the proportion of R2 resections was much higher in the D2 group (13.6% vs. 2.7%; *p* = 0.001). After excluding non-curative resections, the final analysis was based on data concerning 301 subjects, 100 (33.2%) of whom underwent D2 lymphadenectomy, and 201 (66.8%) D3 lymphadenectomy. None of these patients had received preoperative chemotherapy as during the study period the standard of treatment for resectable gastric cancer at our center was up-front surgery”.

The D2 and D3 lymphadenectomies were performed in accordance with the second English edition of the Japanese Classification of Gastric Carcinoma [8]. Tumor invasion (pT) and lymph node status (pN) were recorded using the seventh edition of the UICC pathological tumor node metastasis (pTNM) criteria [9], and histological types were classified as intestinal or mixed/diffuse in accordance with Laurén’s classification [10].

The D3 lymphadenectomies include “therapeutic” interventions (performed when nodal metastases in the third level stations had been clinically detected) and “prophylactic” interventions.

The IRB of Verona University approved this retrospective study (approval details are: Prot. DBCES001-Project 2175CESC-08th May 2019).

## 3. Statistical Analyses

The significance of the differences in quantitative and categorical variables between the D2 and D3 group was respectively evaluated using the non-parametric Wilcoxon-Mann-Whitney rank-sum test and Fisher’s exact test. Survival was estimated using Kaplan-Meier curves, with post-operative deaths and deaths due to recurrence being considered terminal events, and deaths due to other causes being censored at the time of their occurrence. The survival curves were compared using the log-rank test when the hazard functions were proportional over time or the Wilcoxon-Breslow-Gehan test when they were not [11].

The impact of the extent of lymphadenectomy on disease-related survival (DRS) was evaluated using a Cox regression model controlling for sex, age, tumor site, Laurén histotype, and T and N stage. The proportional hazard assumption was checked using graphical methods [12] and the interactions between lymphadenectomy and each of the other variables included in the Cox model was also tested.

The statistical analyses were made using Stata statistical software, release 14 (StataCorp, College Station, TX, USA), and statistical significance was set at *p* < 0.05.

## 4. Results

### 4.1. Surgeons’ Propensity to Perform D2 or D3

As shown in Table 1, the patients undergoing D3 lymphadenectomy were significantly younger but, interestingly, there were no significant between-group differences in the T and N stages, tumor site, or Laurén histology. Moreover, D2 lymphadenectomy was more frequently associated with sub-total gastrectomy, and D3 with total gastrectomy.

### 4.2. Short-Term Outcomes

The median number of retrieved nodes was 29 (interquartile range: 24.5–39) after D2, and 43 (34–52) after D3; the median number of positive nodes was also higher after D3 (5, 0–12 vs 3.5, 0–9.5; *p* = 0.039).

PANs were retrieved from nearly all of the patients undergoing D3 (198/201, 98.5%), and from none of the patients undergoing D2; PAN invasion was detected in 30 (15.2%) of the 198 patients, with 16 stations explored after D3. The proportion of PAN invasion was similar in the pT2 (5/37 = 13.5%), pT3 (7/49 = 14.3%) and pT4 (13/91 = 14.3%) tiers, while not significantly increasing in the pT4b tier (5/21 = 24%) (Fisher’s exact test: *p* = 0.718).

In-hospital mortality was slightly higher after D2 (4/100, 4.0% vs 3/201, 1.5%), but the difference was not significant (*p* = 0.226); the same was true of 90-day mortality (7/100, 7% vs 6/201, 3%; *p* = 0.133)

### 4.3. Survival

Total follow-up was 1440 person-years, and the median follow-up of the surviving patients was 122 months (range 40–235).

**Figure 1:**
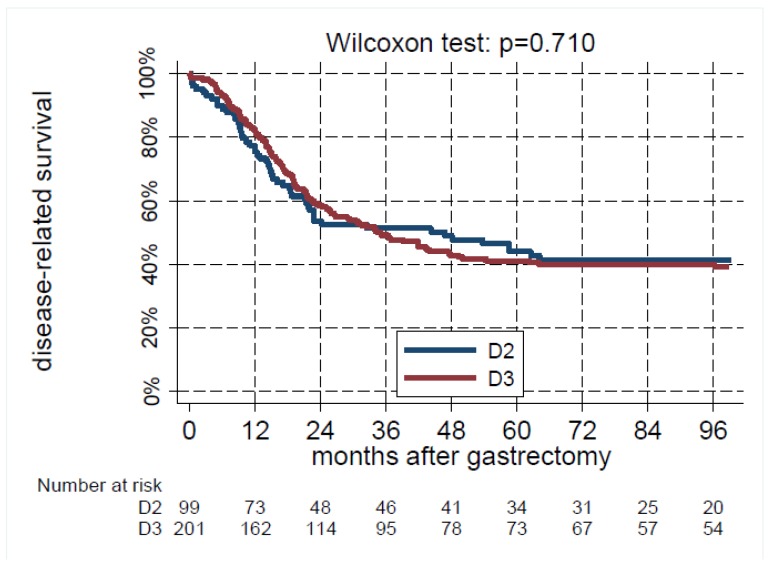
Disease-related survival as a function of the extension of lymphadenectomy, estimated using the Kaplan-Meier method.

Disease-related survival (DRS) was not significantly different between the groups (Wilcoxon test: *p* = 0.710). The 5-year DRS rate was 44% (95% CI 34–54%) after D2 and 41% (34–48%) after (Figure 1).

As expected, the survival rate was quite low in the 30 PAN+ patients: their 3-year survival rate was 19% (95% CI 7–35%) against the 54% (46–61%) of the other patients undergoing D3.

**Figure 2:**
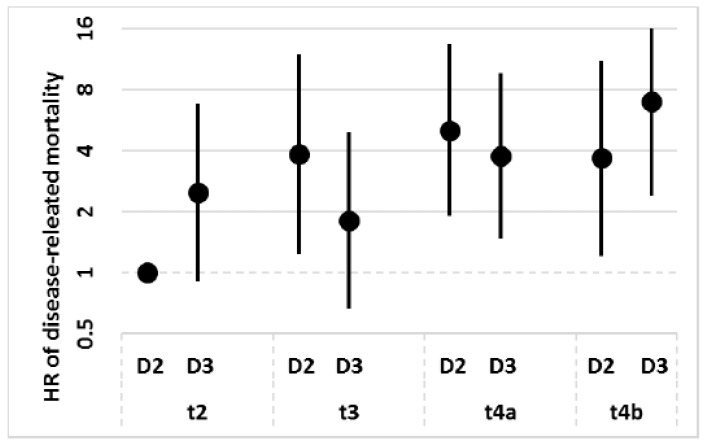
Hazard ratios (HRs) of disease-related deaths as a function of T tier and the extension of lymphadenectomy. The HRs were derived from a Cox regression model controlling for sex, age, tumor site, Laurén histotype, and N stage. The points are HRs and the bars are their 95% confidence intervals.

Multivariable survival analysis showed that the risk of cancer-related death was virtually the same after D2 or D3 (Hazard Ratio (HR) of D3 vs D2 = 0.97, 95% CI 0.67–1.42; *p* = 0.893). However, there was a significant interaction between the depth of tumor invasion and extent of the lymphadenectomy (log-rank test: *p* = 0.012). As shown in Figure 2, prognosis was better after D2 among T2 and T4b patients, but better after D3 among T3 patients and (to a lesser extent) T4a patients.

## 5. Discussion

The findings of this retrospective observational study indicate that D3 is not associated with a better prognosis than that of D2 lymphadenectomy in patients with advanced gastric cancer. The relationship between the extent of the lymphadenectomy and DRS significantly varied across pT tiers, with long-term prognosis seeming to be better after D2 in T2 and T4b patients, and better after D3 in T3 patients. A tentative explanation of this changing pattern could be that the costs of super-extended lymphadenectomies exceed the benefits in less advanced cancers (pT2), while the D3 procedure becomes useless in very advanced cases such as pT4b when cancer cells have already invaded the surrounding organs and a more aggressive nodal dissection does not enable a better local control of the disease.

The debate concerning the role of “prophylactic” super-extended lymphadenectomy apparently came to an end after the publication of the JCOG 9501 trial that found no survival advantage when D2 lymphadenectomy was extended to PANs in patients with T2b, T3, and T4 gastric cancer [3]. Consequently, prophylactic D2 plus PAN dissection is no longer recommended as a first-choice treatment for patients with curable gastric cancer in the Japanese guidelines [13]. However, it should be remembered that the baseline prevalence of 16 metastases in that trial was rather low (8.5%), probably because it only enrolled patients without macroscopic metastases to PANs, and the control group underwent D2 lymphadenectomy extended to the posterior nodal stations (12p, 13, and 14v), which are not usually resected in the case of a conventional D2 [14].

Furthermore, although the trial did not find that PAN dissection led to any significant survival advantage in comparison with D2 alone in the sample as a whole, it is important to note that it did highlight significant interactions between the extent of the lymphadenectomy and T (*p* = 0.004) or N status (*p* = 0.003): patients with less advanced cancer (T2b and N0) showed a significant benefit from PAN dissection [3]. Indeed, the authors tested eleven interactions, which did not represent the primary endpoint of the trial. In our opinion, however, the interactions between the extent of the lymphadenectomy and T or N status cannot be dismissed as a bias due to multiple testing because they were highly significant and consistently involved the most important prognostic variables.

Interestingly, like those of the JOCG trial, our findings also indicate that D3 lymphadenectomy might be associated with better DRS in patients with tumors with subserosal invasion, which were classified as T2b tier in the JCOG trial according to the sixth edition of the TNM and as T3 tier in our study according to the seventh edition.

A recent Japanese phase II trial [6] has provided indirect support in favor of PAN dissection in the presence of extensive lymph node metastases. The patients who had bulky nodes at D2 stations and/or positive PANs upon clinical examination underwent aggressive treatment, including two courses of neo-adjuvant chemotherapy followed by PAN dissection, and those with bulky nodes at the D2 stations, or positive PANs alone had outstanding 3-year survival rates of respectively 76% and 64%, whereas the 3-year survival rate of those with both of these characteristics was only 17% [6]. Of note, in this trial PAN dissection in patients with bulky nodes at the D2 stations only is a prophylactic procedure [6]. 

The possible prophylactic role of PAN dissection is also indirectly supported by a Korean study of 2618 patients who underwent gastrectomy and D2 lymphadenectomy for gastric cancer [15]. Five years after surgery, loco-regional relapses had occurred in only 8.5% of cases, and these were mainly observed outside the dissected area (90.4%), particularly stations 16a2 (46%) and 16b1 (60%). The most likely explanation is that some of these patients with advanced gastric cancer had PAN metastases that were not detected by baseline preoperative imaging. Whether these patients would have benefitted from prophylactic PAN dissection could be a matter of debate.

In this era of tailored multi-modal treatment, it is worth verifying whether PAN dissection may be indicated in specific patient subgroups. A previous study by our group [16] found that loco-regional recurrences were significantly fewer after D3 than after D2 in the subgroup of advanced gastric cancer patients with a highly lymphotropic mixed/diffuse histotype. The interaction between D2/D3 and Lauren histology was not replicated in the present study, when considering survival rather than gastric cancer recurrence. However, it should be reminded that histology seems to have a delayed effect on survival [12].

It is of course necessary to consider the risk of post-operative complications and mortality after a super-extended lymphadenectomy, and so D3 procedures should only be performed in dedicated high-volume hospitals where they can be performed safely, as demonstrated by some of our previous findings [17].

The present study has several strengths. It is based on one of the largest Western series of patients undergoing extended or super-extended lymphadenectomy; the cases were consecutively and prospectively enrolled; and none of the patients had received neo-adjuvant chemotherapy, which provided a unique opportunity to study lymph node status after super-extended lymphadenectomy without previous down-staging.

The main limitation of the study is that its observational nature means that an indication bias cannot be ruled out. The patients undergoing D3 lymphadenectomy were significantly younger and, although the two groups were similar in terms of the main risk factors considered (T and N stage, tumor site and Laurén histology), the influence of unknown confounders cannot be excluded, even in multivariable analysis. Another limitation is the lack of standardized adjuvant treatments throughout the study period, which may have led to an improper assessment of its potential impact on survival and the risk of recurrence.

In conclusion, ten years have passed since the negative results of the JCOG 9501 trial were published, but we do not think that the debate about prophylactic super-extended lymphadenectomy has actually come to an end. The JCOG trial showed that D3 lymphadenectomy can be beneficial in patients with T3 cancer but, as it was beyond the scope of the confirmatory analysis, this collateral finding was not included in the trial abstract or conclusions. Accordingly, our observational study detected a significant interaction between extended lymphadenectomy and T tier, with improved survival after D2 in T2 and T4b cancers, and after D3 in T3 cancers.

In our opinion, the findings of the present study are sufficient to justify the randomized controlled trial comparing standard D2 and super-extended lymphadenectomy after neoadjuvant treatment in patients with advanced gastric cancer without clinically detectable PAN involvement that has just started in Italy (ClinicalTrials.gov Identifier: NCT03961373). Given our previous finding that loco-regional recurrences are significantly fewer after D3 than after D2 in the subgroup of advanced gastric cancer patients with a mixed/diffuse histotype [16], the results of the ongoing randomized trial will be stratified according to Laurén /WHO histotypes.

## 6. Conclusions

The findings of this study suggest that D3 lymphadenectomy is not routinely indicated for patients with advanced gastric cancer, although differences in survival after D3 across T tiers deserve further consideration and may help to identify those patients who may benefit from prophylactic PAN dissection in the era of multimodal treatment.

## Figures and Tables

**Table 1:** Main demographic and clinical characteristics of the patients at the baseline. The bold highlights the significant differences.

	No.	D2 Lymphadenectomy *n* = 100 (33.2%)	D3 Lymphadenectomy *n* = 201 (66.8%)	*p*-Value
Sex				0.799
Male	193	63 (32.6%)	130 (67.4%)	
Female	108	37 (34.3%)	71 (65.7%)	
Mean age ± SD (years)		69.8 ± 12.3	62.2 ± 10.7	<0.001
Tumor site				0.206
Siewert III	28	10 (35.7%)	18 (64.3%)	
Fundus	65	16 (24.6%)	49 (75.4%)	
Body	77	23 (29.9%)	54 (70.1%)	
Antrum	131	51 (38.9%)	80 (61.1%)	
Histology (Laurén)				0.622
Intestinal	174	56 (32.2%)	118 (67.8%)	
Mixed/diffuse	126	44 (34.9%)	82 (65.1%)	
Pathological T stage				0.133
pT2	69	29 (42.0%)	40 (58.0%)	
pT3	65	16 (24.6%)	49 (75.4%)	
pT4a	132	41 (31.1%)	91 (68.9%)	
pT4b	35	14 (40.0%)	21 (60.0%)	
Pathological N stage				0.153
pN0	77	35 (45.5%)	42 (54.5%)	
pN1	42	12 (28.6%)	30 (71.4%)	
pN2	61	18 (29.5%)	43 (70.5%)	
pN3a	67	19 (28.4%)	48 (71.6%)	
pN3b	54	16 (29.6%)	38 (70.4%)	
Type of gastrectomy				<0.001
Proximal	16	9 (56.3%)	7 (43.7%)	
Total	167	33 (19.8%)	134 (80.2%)	
Sub-total	114	54 (47.4%)	60 (52.6%)	
Other	4	3 (100%)	-	
